# Chronic Malarial Toxemia, Its Prevalence in New York City—Causes and Treatment by Alteratives

**Published:** 1897-08

**Authors:** J. P. Sheridan

**Affiliations:** New York


### CHRONIC MALARIAL TOXEMIA, ITS PREVALENCE IN NEW YORK CITY—CAUSES AND TREATMENT BY ALTERATIVES.

BY J. P. SHERIDAN, M. D., NEW YORK.

It is not my intention here to dwell upon the many manifestations presented in chronic malarial toxemia, nor to appeal to the evidence of medical statistics in proof of the great number of these cases prevalent in New York City. The numerous excavations necessary in the erection of buildings, the turning over of soil in cellars long hidden from sunlight, the blasting of rock, etc., etc., are prime factors in establishing conditions which develop the activity of a germ whose influence all physicians are sooner or later called upon to combat. We do not know positively when or in what form the pathogenic organisms exist outside of the body. We are equally uncertain as to their mode of entrance, most authorities looking on the respiratory organs as principally or exclusively concerned. Be this as it may, symptoms present themselves which do not yield to quinine, nor to Wurburg's Tincture, except that they are palliated, not cured, recurring again and again, and the same result being attained and a like exacerbation occurring to the individual upon a new exposure. No one can deny that quinine is a *sine qua non* in the treatment of malaria, when it is administered during the intermission or remission of an acute exacerbation, but it is in those types which belong to the chronic form, and especially the anemia present, which I wish to call attention to.

In the treatment of these conditions I have used Barclay's solution of bromide of gold and arsenic (Arsenauro) with the most gratifying results. My attention has been repeatedly called to this solution, and its companion, liquid bromide gold, arsenic and mercury (Mercauro), but like most of my colleagues I did not realize their efficacy until I had put them to a crucial test. This I have done in a large number of cases, extending over a period of twelve months. At first I made the common error of discontinuing their use too soon. We must push them as we do iodide of potassium—to the point of toleration. In persons who could not take Fowler's Solution these combinations, as presented, were readily borne, the irritant effect of the arsenic being overcome. It is well to administer these solutions largely diluted, giving them in a half goblet of water if possible, and keeping the patient near the point of toleration for at least six weeks. Preference should be given to the mercurial combination in malarial anemia and malarial cachexia and splenic enlargement. In masked intermittents, or "malaria larvata," quinine is useless, but I have demonstrated to my satisfaction the value of liquid *arsenii et*

*auri* bromide (Arsenauro). I have recently used these solutions in other conditions requiring alteratives and my results have been most satisfactory.

I particularly emphasize the point that these solutions must be continued for a reasonable length of time. In many cases improvement does not manifest itself promptly. Arsenauro and Mercauro are in no wise palliatives. One bottle may apparently give no result. Palliatives never affect structures, but only functions. The organic changes remain just the same, no matter how long palliatives are administered. "This is according to the therapeutic law to which there are no exceptions, that any drug whose specific medicinal effects can be secured by one dose (or a few doses) can not modify or affect a structural change." I refer to these principles of therapeutics here because I wish to emphasize my assertion that these solutions are not palliative, but curative.—*The Times and Register, May 29th, 1897.*

